# T187. ALTERED DNA METHYLATION OF THE OXYTOCIN RECEPTOR GENE IS ASSOCIATED WITH SUSCEPTIBILITY TO PSYCHOSIS AND ANHEDONIA-ASOCIALITY IN FEMALES: EPIGENETIC EVIDENCE IN RECENT-ONSET SCHIZOPHRENIA AND ULTRA-HIGH RISK FOR PSYCHOSIS

**DOI:** 10.1093/schbul/sby016.463

**Published:** 2018-04-01

**Authors:** Minji Bang, Se Joo Kim, Kyung Ran Kim, Su Young Lee, Jin Young Park, Eun Lee, Jee In Kang, Suk Kyoon An

**Affiliations:** 1 Yonsei University College of Medicine; 2 Dankook University College of Medicine

## Abstract

**Background:**

Oxytocin is one of the key hormones involved in human social and emotional processing. In this regard, abnormal functioning of the oxytocin system has been suggested to influence on the clinical manifestation of schizophrenia, especially negative symptoms. The aim of the present study was to investigate epigenetic modification of the oxytocin receptor gene (OXTR) and its association with negative symptoms in individuals with recent-onset schizophrenia (SCZ) and at ultra-high risk (UHR) for psychosis.

**Methods:**

Sixty-four SCZ patients (< 5 years of duration of illness; 25 men, 39 women), 46 UHR individuals (27 men, 19 women), and 98 healthy controls (HCs; 46 men, 52 women) participated in the present study. DNA methylation was quantified from peripheral blood using pyrosequencing at CpG sites in OXTR intron 1 (hg19, chr3: 8,810,729–8,810,845) and exon 3 (hg19, chr3: 8,809,281–8,809,534). The severity of negative symptoms in clinical groups was measured using the Scale for the Assessment of Negative Symptoms (SANS) and Scale for the Assessment of Positive Symptoms (SAPS).

**Results:**

A multivariate analysis of covariance revealed significant differences in OXTR methylation between groups (F = 16.00, p < 0.001) and gender (F = 2.84, p = 0.025). Compared to HCs, both UHR and SCZ participants showed lower levels of OXTR intron 1 methylation, particularly at CpG site -934 upstream of the OXTR start codon (HCs = 47.3 ± 4.1 [mean ± SD], UHR = 38.8 ± 4.8, SCZ = 40.2 ± 5.3; F = 73.74, p < 0.001). Besides, female participants showed higher OXTR intron 1 methylation at CpG site -934 than male participants (male = 42.3 ± 6.1, female = 44.1 ± 5.8, F = 9.08, p = 0.003). Multiple linear regression analysis with clinical symptoms demonstrated that the degree of DNA methylation at CpG site -934 was significantly associated with the SANS anhedonia-asociality subscale scores in the entire group of female UHR and SCZ participants (beta = -0.44, p = 0.001).

**Discussion:**

The present study demonstrated decreased OXTR methylation in both UHR and SCZ individuals compared to HCs. Furthermore, the severity of anhedonia-asociality was significantly associated with the degree of OXTR methylation in female UHR and SCZ individuals. These findings suggest that epigenetic aberration of OXTR may confer susceptibility to schizophrenia spectrum psychosis and influence the early pathogenesis of schizophrenia prior to the onset of overt psychosis, particularly in females.

